# Divergent populations of HIV-infected naive and memory CD4^+^ T cell clones in children on antiretroviral therapy

**DOI:** 10.1172/JCI188533

**Published:** 2025-03-06

**Authors:** Mary Grace Katusiime, Victoria Neer, Shuang Guo, Sean C. Patro, Wenjie Wang, Brian Luke, Adam A. Capoferri, Xiaolin Wu, Anna M. Horner, Jason W. Rausch, Ann Chahroudi, Maud Mavigner, Mary F. Kearney

**Affiliations:** 1 HIV Dynamics and Replication Program, Center for Cancer Research, National Cancer Institute (NCI), NIH, Frederick, Maryland, USA.; 2 Cancer Research Technology Program and; 3 Advanced Biomedical Computational Science, Leidos Biomedical Research Inc., Frederick National Laboratory for Cancer Research, Frederick, Maryland, USA.; 4 Department of Pediatrics, Emory University School of Medicine, Atlanta, Georgia, USA.

**Keywords:** AIDS/HIV, Virology, Adaptive immunity, Cellular immune response, T cells

## Abstract

**BACKGROUND:**

Naive cells comprise 90% of the CD4^+^ T cell population in neonates and exhibit distinct age-specific capacities for proliferation and activation. We hypothesized that HIV-infected naive CD4^+^ T cell populations in children on long-term antiretroviral therapy (ART) would thus be distinct from infected memory cells.

**METHODS:**

Peripheral blood naive and memory CD4^+^ T cells from 8 children with perinatal HIV on ART initiated at age 1.7–17 months were isolated by FACS. DNA was extracted from sorted cells, and HIV proviruses were counted, evaluated for intactness, and subjected to integration site analysis (ISA).

**RESULTS:**

Naive CD4^+^ T cells containing HIV proviruses were detected in children with 95% statistical confidence. A median 4.7% of long terminal repeat–containing naive CD4^+^ T cells also contained HIV genetic elements consistent with intactness. Full-length proviral sequencing confirmed intactness of 1 provirus. In the participant with the greatest degree of naive cell infection, ISA revealed infected expanded cell clones in both naive and memory T cells, with no common HIV integration sites detected between subsets. Divergent integration site profiles reflected differential gene expression patterns of naive and memory T cells.

**CONCLUSION:**

These results demonstrate that HIV persisted in both naive and memory CD4^+^ T cells that underwent clonal expansion and harbored intact proviruses, and suggest that infected memory T cell clones do not frequently arise from naive cell differentiation in children with perinatal HIV on long-term ART.

**FUNDING:**

Center for Cancer Research, NCI; Office of AIDS Research; NCI FLEX; Children’s and Emory Junior Faculty Focused Award.

## Abstract 2

Infected naive CD4+ T cells comprise a significant fraction of the viral reservior in children with HIV on antiretroviral therapy.

## Introduction

Of the 39.9 million people living with HIV (PLWH) in 2023, 1.4 million were children under 15 years of age (1), most of whom acquired the virus through vertical transmission during the mother’s pregnancy, labor, delivery, or breastfeeding (2). Although maternal use of antiretroviral therapy (ART) is highly effective in preventing vertical HIV transmission (3), new pediatric infections continue to occur due largely to the high prevalence and incidence of HIV in women of childbearing age, unavailable or incomplete implementation of prevention modalities, and/or suboptimal timing of or adherence to ART (4).

Without treatment, more than half of children who acquire HIV vertically die by 2 years of age (5), whereas the median survival time for ART-naive adults with HIV is 11 years (6). Children living with HIV (CLWH) also tend to have higher peak viremia and take longer to reach the setpoint compared with adults (7, 8). Moreover, although early ART administration clearly benefits both adults and children (9–16), anecdotal reports suggest that post-treatment control in children may be more attainable in the setting of ART initiated shortly after birth (17–19).

These distinctive features of perinatal HIV infection are attributable, at least in part, to the equally distinctive characteristics of the developing immune system. For instance, in healthy individuals, naive cells comprise a much greater fraction of the CD4^+^ T cell pool early in life relative to adulthood, declining from approximately 90% at birth to approximately 50% by age 40 years (20). Neonatal naive T cell populations are also derived from different stem cells than those in adults, have less T cell receptor (TCR) diversity than adult naive T cells, are less prone to contract into memory cells following activation, and are more likely to proliferate homeostatically, particularly under lymphopenic conditions (21–24).

Although memory cells comprise the greatest proportion of CD4^+^ T cells with HIV proviruses in adults on ART (25, 26), HIV proviruses are also found in naive CD4^+^ T cells (26–34). The primary block to HIV infection of naive CD4^+^ T cells occurs at the binding stage of the virus life cycle (35, 36), as the cellular chemokine receptor CCR5, which functions as an HIV entry coreceptor, is not well expressed in this cell type. The alternate HIV coreceptor CXCR4 is present on naive CD4^+^ T cells, but viruses that utilize this coreceptor are typically not associated with transmission. Given the dominance of naive CD4^+^ T cells at the time of vertical HIV acquisition, we hypothesized that the resulting pool of persistently infected naive CD4^+^ T cells is important to consider in studies of curative interventions for CLWH, and we thus sought to more deeply elucidate features of the naive T cell reservoir.

We have previously demonstrated approximately 1% HIV proviral intactness frequency in PBMCs from children who started ART at a median age of 5.4 months (37), and integration site analysis (ISA) showed the early emergence of clonally infected cell populations in CLWH (38). Moreover, a comprehensive 2-year longitudinal analysis of provirus intactness frequencies, integration sites, and immunological consequences in treated and untreated CLWH showed that immediate ART administration in neonates can reduce the reservoir size and induce a distinct immune profile (39). While each of these studies revealed features of genetic structures of provirus cell populations, sites of integration, and/or a propensity toward clonal expansion in children on ART, none addresses how these properties might differ in CD4^+^ naive and memory T cell subsets.

In the present work, we characterize the proviruses that persisted in circulating naive and memory CD4^+^ T cells sorted from 8 CLWH who received ART early in life. Using multiple displacement amplification (MDA), an adapted intact proviral DNA assay (IPDA), and ISA, we provide evidence for the presence of intact proviruses in and clonal expansion of infected naive CD4^+^ T cells of CLWH on long-term ART, including in samples from 1 participant in whom the fractional contribution of naive T cells to the infected cell population at the time of sampling was estimated to be 40%. In-depth analysis of samples from this participant detected no overlap between infected cell clonal populations in naive and memory CD4^+^ T cells. Additionally, proviral integration sites detected in infected naive cells were more often found in genes expressed at higher levels in that subset relative to levels in the memory cell subset. However, except for the most highly expressed genes, this pattern was not observed among infected memory cell integration sites, perhaps indicative of persistent infected naive cells being activated and differentiating into memory cells in the years prior to sample collection. Taken together, our findings demonstrate that naive CD4^+^ T cells are an important and distinct reservoir in children with perinatally acquired HIV.

## Results

### Participants, samples, and cell sorting.

Four male and 4 female children, aged 5–11 years, were enrolled in the study. All 8 children acquired HIV vertically, were on ART, and had levels of HIV RNA in plasma that were fewer than 20 copies/mL at the time of blood collection. The demographic and virologic characteristics of the participants are shown in Table 1. The HIV subtype was known for 7 of the 8 children, and 5 children had subtype B, while 2 of the children acquired subtype C. The children were initiated on ART at a median age of 8.75 months (range, 1.5–17 months) and had received ART for a median of 8 years (range, 5–10 years). Populations of naive and memory CD4^+^ T cells were isolated from PBMCs by FACS, with naive CD4^+^ T cells defined as CD45RO^–^CD45RA^+^CD28^+^CD27^+^CCR7^+^CD95^–^ and memory CD4^+^ T cells as CD45RO^+^CD95^+^ (Supplemental Figure 1; supplemental material available online with this article; https://doi.org/10.1172/JCI188533DS1). Sorting gates were set far apart to minimize contamination, and the median purity of the sorted cells was 97% (range, 93%–100%; Table 2 and Supplemental Table 1).

### HIV DNA persists in naive CD4^+^ T cells in CLWH on long-term ART.

**Figure 1:**
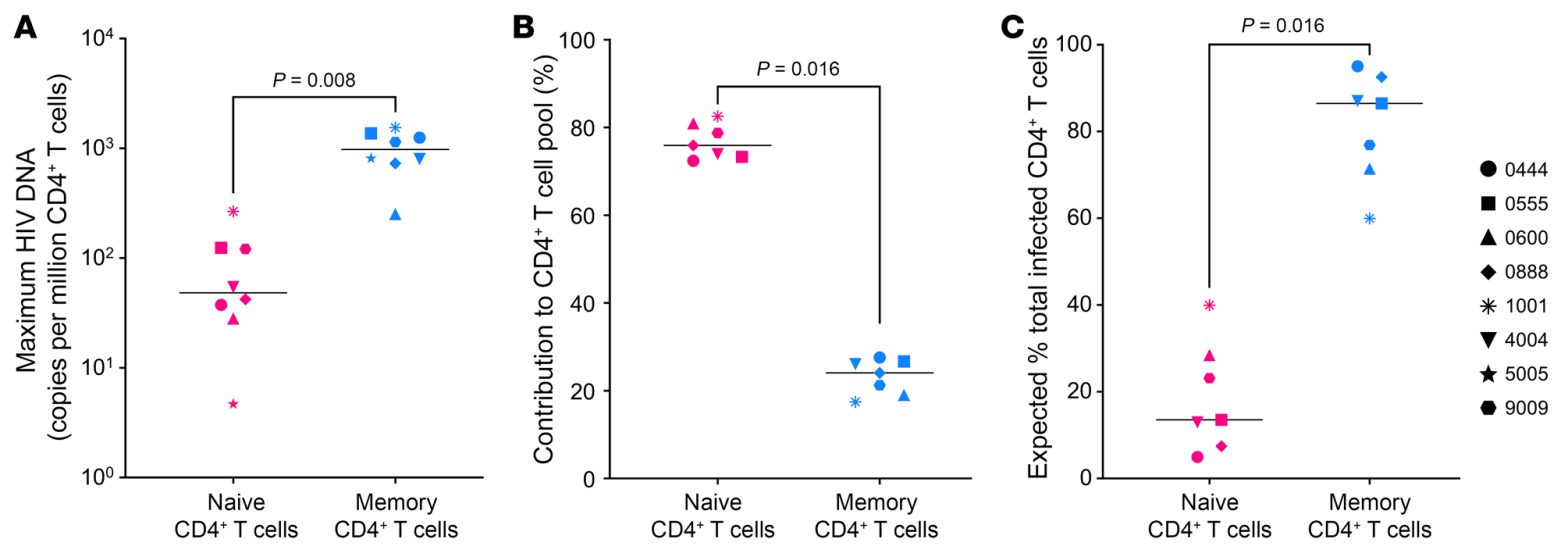
HIV persistence in peripheral naive and memory CD4^+^ T cells from CLWH. (**A**) Frequency of HIV DNA in sorted populations of naive and memory CD4^+^ T cells. (**B**) Relative contribution of naive and memory CD4^+^ T cells to the CD4^+^ T cell pool assessed by flow cytometry. (**C**) Relative contribution of naive and memory CD4^+^ T cells to the total HIV reservoir in CD4^+^ T cells calculated after correction for potential contamination of the sorted cells. Horizontal bars represent the median.

Cellular DNA, including HIV proviruses, was extracted from sorted cell populations, dispensed onto microtiter plates at limiting proviral dilutions, and amplified by MDA as described in Methods and in a previous publication (40). This approach enabled resampling of the amplified products for subsequent analyses including real-time PCR screening, ISA, and proviral sequencing. To calculate the infected cell frequencies in the naive and memory CD4^+^ T cell sorts, MDA products were screened by PCR for the presence of the HIV long terminal repeat (LTR). Poisson-corrected LTR^+^ well counts were divided by the total numbers of cells screened. HIV LTR DNA was detected in naive CD4^+^ T cell sorts from all participants, with a median frequency of 48 (range, 5–266) copies per million cells (Table 2 and Figure 1A). HIV LTR DNA was detected in the memory T cell sorts, with a median of 977 (range, 252–1,546) copies per million cells (Supplemental Table 1 and Figure 1A).

To assess the robustness of these data, we then estimated the frequency of possible contaminating memory CD4^+^ T cells in the sorted naive cells. For each participant, the expected and maximum numbers of possible memory T cell contaminants (95% confidence) within the sorted naive CD4^+^ T cells were calculated using the number of cells, the number of HIV LTR copies, and sort purities as previously described (41) (Table 2). In turn, these values were used to calculate minimum and expected HIV LTR copies arising from naive cells, rather than contaminating memory cells. The median number of HIV-infected naive cells, after correcting for possible contaminating memory cells, was 33 per million (Table 2). The corresponding values were also calculated for the memory T cell sorts, resulting in a median of 975 HIV-infected memory cells per million (Supplemental Table 1).

Using HIV-infected cell frequencies corrected for possible cross-contamination and frequencies of naive and memory CD4^+^ T cells obtained from the cell sorting (Figure 1B), we calculated the contribution of each subset to the total HIV-infected peripheral CD4^+^ T cell population (Figure 1C). Participant 5005 was excluded from this analysis because the observed Poisson-corrected measured number of infected naive T cells was less than the number expected from contaminating memory cells in this sort. The contribution of naive CD4^+^ T cells to the total number of HIV^+^ CD4^+^ T cells ranged from 4.9% to 40% across the remaining participants, with a median contribution of 13.5% (Figure 1C).

### Genetically intact proviruses persist in naive CD4^+^ T cells in CLWH on long-term ART.

**Figure 2:**
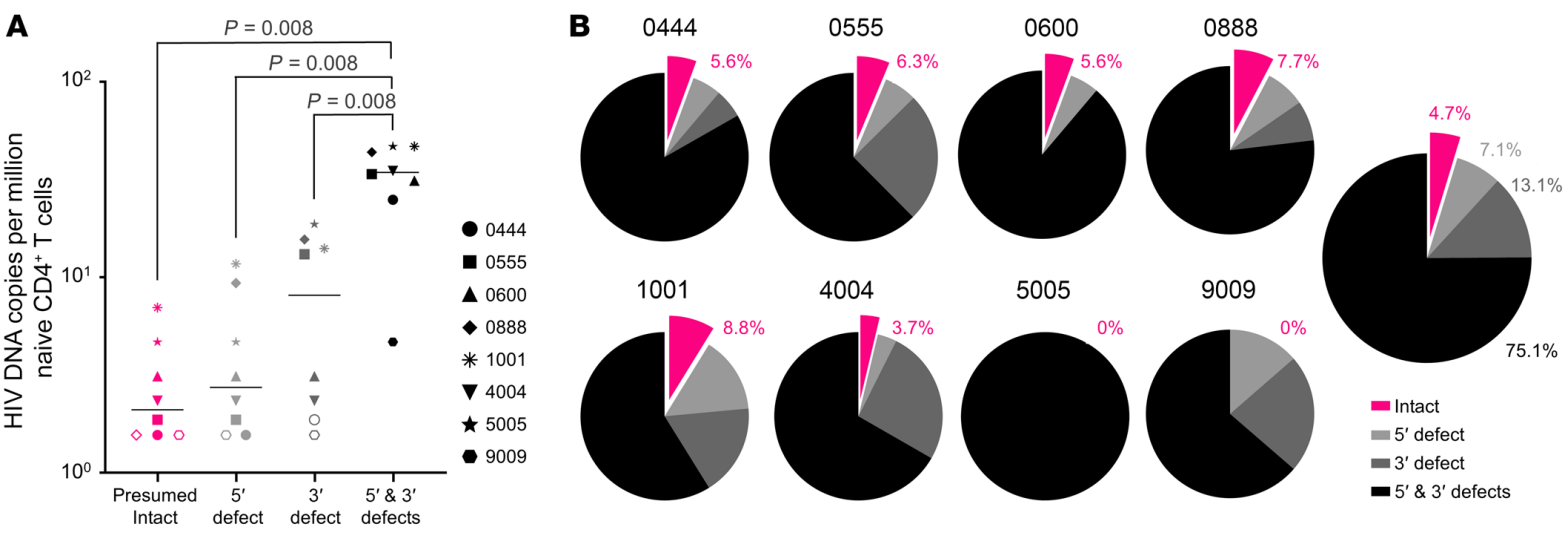
Persistent potentially intact and defective proviruses in naive CD4^+^ T cells from CLWH. (**A**) Levels and (**B**) relative frequencies of potentially intact, 5′-defective, 3′-defective, and 5′- and 3′-defective HIV proviruses estimated by an IPDA. Open symbols indicate undetectable values with the limit of detection set based on cell input. Horizontal bars represent the median. Wilcoxon matched-pairs signed rank test were used to determine statistical significance.

To assess whether any of the HIV LTRs detected in naive CD4^+^ T cells derived from intact proviruses, we screened HIV LTR^+^ MDA products for HIV Psi (Ψ) and Rev response elements (RREs) by real-time PCR using a modified IPDA (Figure 2). Detection of both HIV genetic elements can be used as a surrogate for full HIV DNA sequencing to infer proviral intactness, as previously reported (42). Of the more than 200 wells containing single infected naive cells across all 8 participants, 13.1% screened positive for LTR^+^ Psi (indicating a 3′-defective HIV), 7.1% screened positive for LTR^+^ RRE (5′-defective), and 75.1% did not screen positive for either Psi or RRE, indicating a provirus with both 5′ and 3′ defects or a solo LTR (43). The 4.7% of MDA-amplified proviruses that screened positive for both Psi and RRE were potentially intact and capable of contributing to rebound viremia if ART were withdrawn. This intactness frequency is comparable to the frequencies previously reported for children and adults with HIV on ART, irrespective of T cell subtype (37, 44).

Having identified 10 candidate-intact proviruses in naive CD4^+^ T cells across all donors, we sought to directly confirm intactness by PCR amplification and near-full-length sequencing. Despite the challenges associated with segmental amplification of HIV proviruses by MDA, as well as the inherent genetic variation of viral genomes (45), we were able to assemble a contiguous full-length sequence for 1 provirus found in the naive CD4^+^ T cell population from participant 1001 (GenBank accession number PV031991). We confirmed sequence intactness using the proviral sequence annotation and intactness test (ProSeq-IT) (45). To ensure that the intact sequence was not a laboratory contaminant, we performed phylogenetic analysis by comparing the U3 and *pol* regions with laboratory strains of HIV, with sequences obtained from other donor samples in the laboratory, and with a random collection of other clinical samples of the same subtype (Supplemental Table 2 and Supplemental Figure 2). The genetic distance between the intact proviral sequence found in naive CD4^+^ T cells from participant 1001 and laboratory-adapted HIV strains indicated that the new HIV sequence was not a laboratory contaminant. Together, our results demonstrate that intact HIV proviruses persisted in naive CD4^+^ T cells in children with vertically acquired HIV on long-term ART.

### Correlations among immunological, clinical, and virological study parameters.

**Figure 3:**
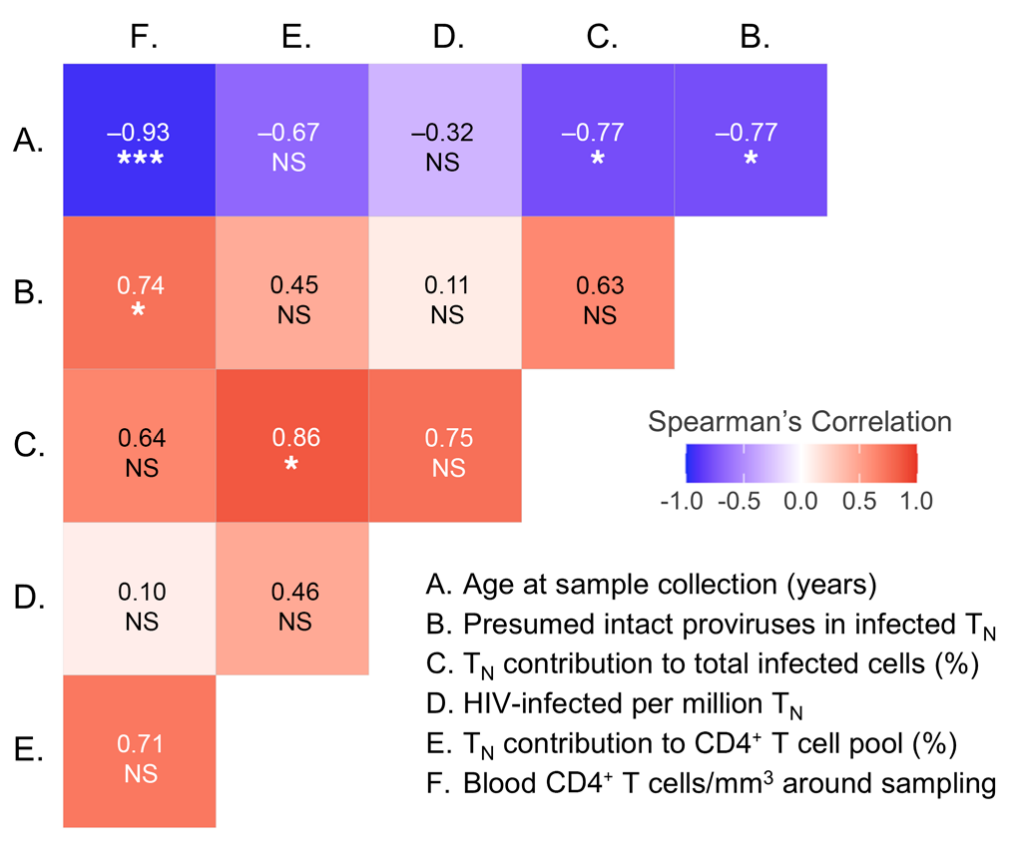
Correlations among immunological, clinical, and virological parameters. Spearman correlations among CD4^+^ T cell counts around the times of sampling, several aspects of infected and uninfected naive CD4^+^ T cells (T_N_), and approximate participant age at the time of sampling. Statistical significance: *P* ≥ 0.05 (NS); *P < 0.05, **P < 0.01, and ***P < 0.001.

Multiple pairwise Spearman correlation analyses revealed significant correlations among immunological, clinical, and virological study parameters (Figure 3). Most notably, naive T cell contributions to peripheral CD4^+^ T cell pools, infected naive T cell contributions to total infected cells, and fractions of proviruses in infected naive T cells that were potentially intact were all negatively correlated with the participant’s age at the time of sample collection (with Spearman correlation coefficients of –0.67, –0.77, and –0.77, respectively). These findings are likely reflective of a natural transition from naive to memory CD4^+^ T cell predominance during normal immune system development, and perhaps also the selective decay of infected naive CD4^+^ T cell populations harboring intact proviruses after years on ART.

### Proviruses persisting in naive CD4^+^ T cells in CLWH on long-term ART are predicted to be CCR5 tropic.

Whereas most transmitted HIVs are R5 tropic (46), naive CD4^+^ T cells express CCR5 only transiently and are relatively resistant to infection with R5-tropic virus in vitro (35). To determine whether HIV^+^ naive CD4^+^ T cells identified in samples from participants in this study were the result of infection with R5-tropic virus or the less-abundant X4-tropic virus, we sequenced and analyzed the *env* V3 loop region amplified by PCR from RRE^+^ MDA products from participants 0444, 0555, and 1001 (GenBank accession numbers PV031992–PV031999). Coreceptor usage was predicted in silico using both the position-specific score matrices (PSSM), which generates probability scores from the identities of amino acids or nucleotides at specific positions (47), and Geno2Pheno (48). All tested proviruses from naive CD4^+^ T cells were predicted to utilize CCR5, in accordance with previously published results for naive CD4^+^ T cells obtained from adults living with HIV on ART (41).

### Proviruses persisting in naive CD4^+^ T cells in CLWH on long-term ART can be clonally expanded and are distinct from memory CD4^+^ T cell proviral populations.

Identifying and cataloging the sites of integration of HIV proviruses in PLWH on ART can be informative of the molecular and subcellular determinants favorable to integration (49, 50), the viral persistence determinants and selection pressures (51), and the mechanisms and degrees of infected cell clonal expansion (52). To characterize the proviral landscape in naive and memory T cell populations, we therefore performed ISA using HIV^+^ MDA products from participant 1001, selected for the high frequency of infection of naive CD4^+^ T cells. Although insufficient to support a comparative analysis of naive and memory cell integration site profiles, 26 additional integration sites identified in naive cells from participants 0444, 0555, and 9009 have been made available in the Retrovirus Integration Database (RID) (53) in connection with this study.

**Figure 4:**
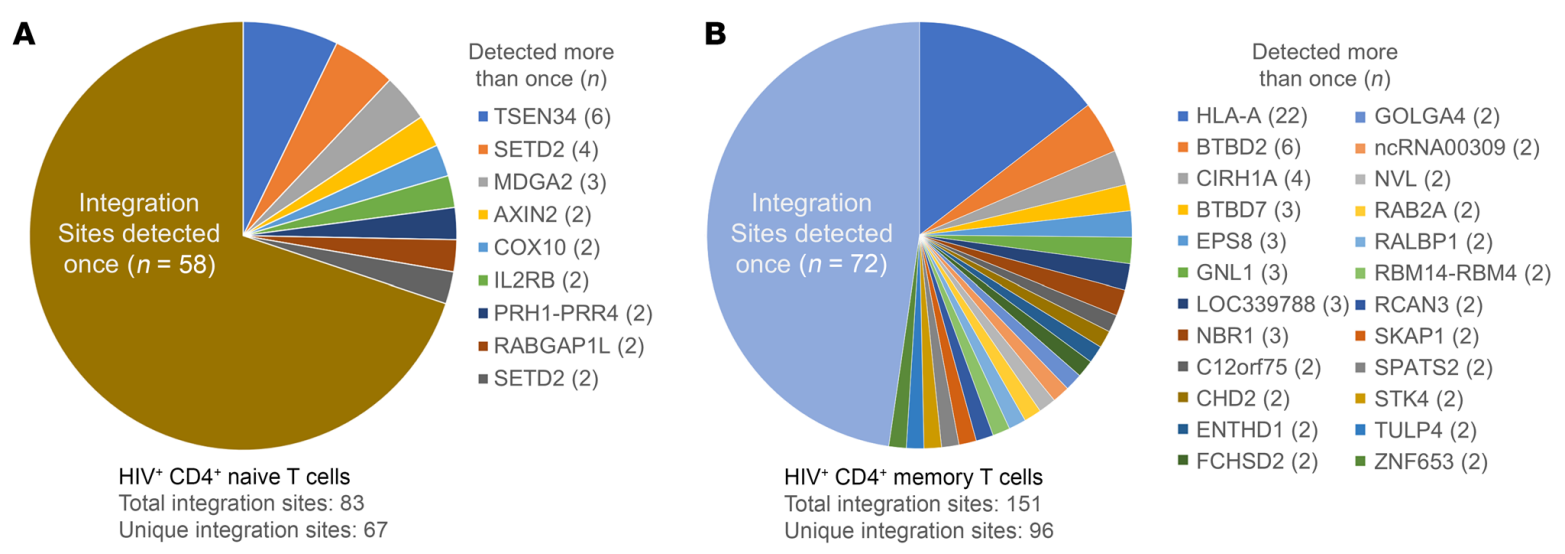
HIV integration site distribution in CD4^+^ T cells from participant 1001. Integration sites identified in (**A**) naive and (**B**) memory CD4^+^ T cells. Total integration sites, the number of unique integration sites, the number of sites detected once, and the specific genes harboring integration sites detected more than once (with number of times detected) in each sorted collection are indicated. Wedge sizes are proportional to the number of integration sites detected once or the number of times specific clonal populations were detected in verified expanded clones.

ISA results for naive and memory CD4^+^ T cells from participant 1001 are shown graphically in Figure 4 and are also available in the RID. We found that 71.7% of the proviral integration sites in naive CD4^+^ T cells were in genes and that 40.3% of proviruses were in the same orientation as the gene. Memory CD4^+^ T cells had similar frequencies of proviral integration sites in genes (71.9%), with 42.7% of proviruses co-oriented with the gene. In naive cells, we identified 67 unique integration sites for 83 MDA-amplified proviruses, 58 of which were each identified exactly once (singlets; 86.5%) and 9 more than once (clones; 13.4%). In memory CD4^+^ T cells, 151 MDA-amplified proviruses yielded 96 unique integration sites, of which 72 were singlets (75%) and 24 were clones (25%). In this context, mapping multiple proviruses to the same integration site definitively established clonal expansion of the harboring T cells. However, it is important to note that the converse, i.e., detecting an integration site only once, did not demonstrate that the T cell harboring that provirus was not part of a clonally expanded population, only that we could neither confirm nor refute this assertion.

Notably, 6 of the 83 (7.2%) amplified proviruses in naive CD4^+^ T cells for which we could identify an integration site mapped to a common location within the *TSEN34* gene. Likewise, 22 of 151 (14.6%) amplified proviruses in memory CD4^+^ T cells mapped to a common integration site in *HLA-A*. These findings are indicative of exceptionally large clonal populations relative to other HIV^+^ cells in this individual. Nevertheless, none of the integration sites identified, including those of proviruses in large clonal populations, were detected in both the naive and memory CD4^+^ T cell subsets.

Taken together, an important implication of these findings is that infected naive and infected memory CD4^+^ T cell pools were not completely mixed. If they were, the binomial probability of finding even the smallest confirmed clone twice in the naive T cell sample (2 of 83) and not at all among memory T cells (0 of 151) would be 0.0251 (or 2.51%). For larger clones, as the number of clonal naive cell integration site detections increases (e.g., 3 of 81 and 4 of 81), the probability of finding none among memory T cells decreases (0.385% and 0.0577%), respectively. Yet none of the clones found in the naive cells were represented among memory cells. The probability of this collective result, assuming complete mixing, equals the product of probabilities calculated for each occurrence individually, or *P* = 2.6086 × 10^–40^, effectively zero. These results demonstrate that naive CD4^+^ T cells in children born with HIV can be infected, can clonally expand, and can persist for years on ART, and that their detection does not result from contamination of infected memory cells in the sorting process.

### Distinct expression patterns of integration site genes in naive and memory CD4^+^ T cells.

**Figure 5:**
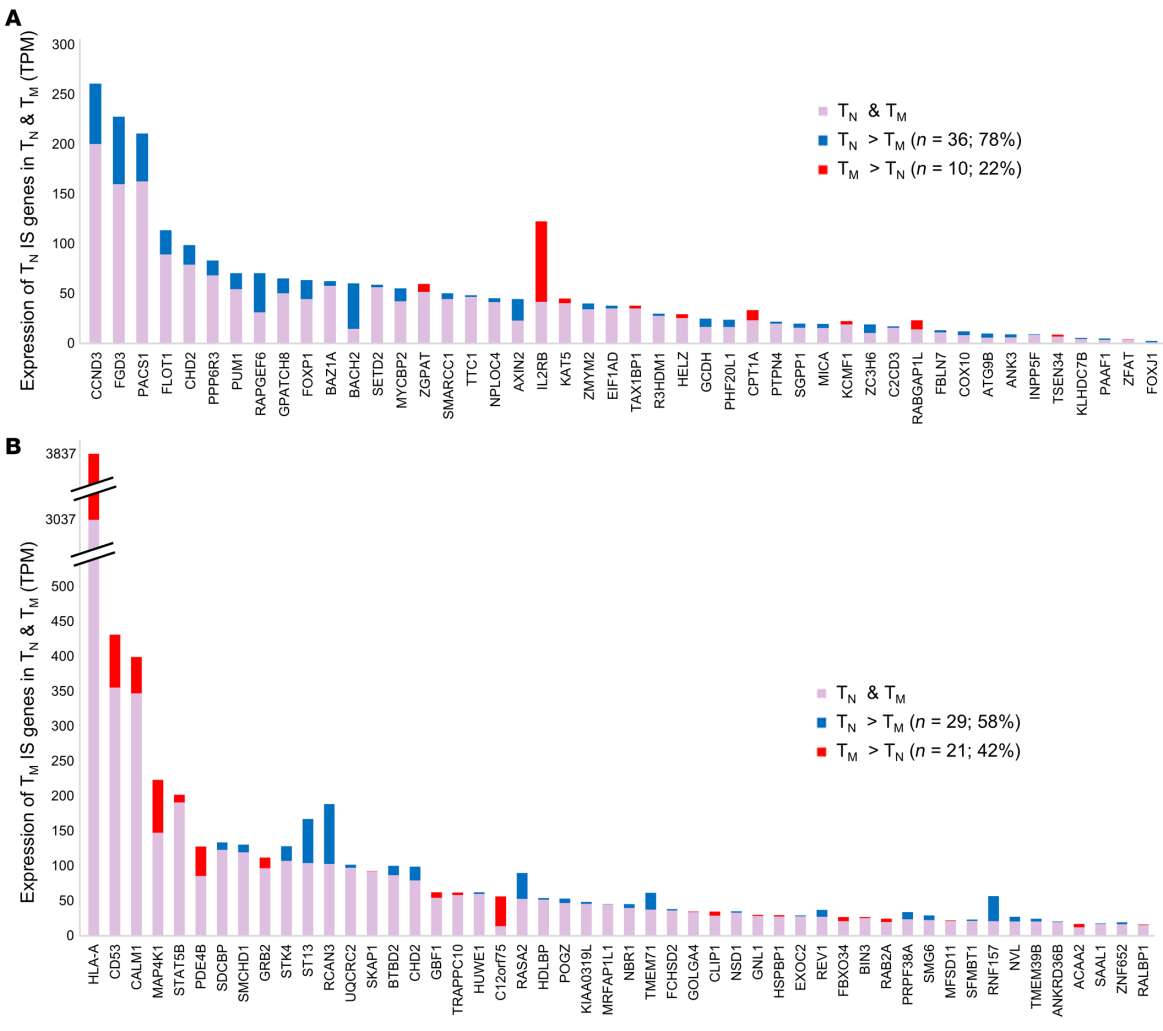
Genic integration sites versus expression levels in naive CD4^+^ and memory T cells. Integration site (IS) genes identified from (**A**) naive and (**B**) memory CD4^+^ T cell sorted collections are listed in descending order of expression levels in the subset in which they were identified (TPM, according to RNA-Seq data). Genes expressed at higher levels in T_N_ are indicated by purple bars with blue caps, the heights of which indicate expression levels in T_M_ and T_N_, respectively. Genes expressed at higher levels in T_M_ are indicated by purple bars with red caps, the heights of which indicate expression levels in T_N_ and T_M_, respectively.

It is now well established that HIV preferentially integrates into expressed genes (49, 50) and that naive and memory CD4^+^ T cells exhibit distinct transcriptional profiles (54). From this foundation, and to explore a potential contributing factor to the sparse overlap among integration site genes identified from sorted naive and memory CD4^+^ T cell subset collections (CHD2 and LOC339788 are the only genes in common, although the integration sites in naive and memory cells are different even in these genes), we examined whether relative expression levels of these genes were generally greater in the CD4^+^ T cell subset in which they were identified. Using a previously published RNA-Seq dataset from sorted peripheral naive and memory CD4^+^ T cells from 8 PWH on ART as a reference (55), expression levels of integration site genes identified in sorted naive and memory CD4^+^ T cells from participant 1001 are presented in Figure 5, A and B, respectively.

All but 1 of the 50 integration site genes identified in naive CD4^+^ T cells had detectable levels of expression in naive T cells, 46 of which yielded average transcripts per million (TPM) values of greater than 2 and are presented in descending order of naive T cell expression levels in Figure 5A. Remarkably, 36 of 46 (78%) naive T cell integration site genes were expressed at to higher levels in naive cells relative to memory cells by a median of 28% (range, 3%–312%). These discrete findings were supported by analyses of relative expression of naive T cell integration site genes in aggregate (Supplemental Figure 3A), i.e., naive T cell integration site genes were expressed in naive CD4^+^ T cells at significantly higher levels than in memory CD4^+^ T cells (*P* = 0.0003, Supplemental Figure 3). The integration site gene that best exemplifies this trend is *BACH2*, with an average relative expression more than 4-fold higher in naive than in memory T cells. Collectively, these results are consistent with the general tendency of HIV to integrate into genes that are more highly expressed. However, this tendency is not absolute, as we found that 10 naive cell integration site genes (22%) were expressed at a median 16% higher levels in memory T cells in the RNA-Seq analysis, including the most notable counterexample *IL2RB*, which is expressed 192% more in memory than in naive T cells.

Yet the tendency for integration site genes to be more highly expressed in the subset in which they were identified was not observed among the 50 most highly expressed integration site genes identified in memory cells, as only 21 (42%) were more highly expressed in memory than in naive T cells (Figure 5B), by a median of 14%. Instead, the majority of memory T cell integration site genes (29 of 50; 58%) were actually expressed at higher levels in naive cells by a median of 15%. These findings are likewise supported by graphical and statistical analyses of memory T cell integration site genes in aggregate (Supplemental Figure 3B), which revealed no statistically significant difference in expression between the 2 subsets (*P* = 0.3435). One potential explanation of these results is that some naive cells that were infected in infancy, collectively harboring proviruses in genes more highly expressed in this subset, were activated and ultimately differentiated into memory during the intervening years on ART prior to sample collection. Such cells, despite collectively having a naive T cell integration site profile, would sort to the memory cell collection and thus mask the trend in this subset.

## Discussion

In this study, HIV proviral populations in circulating naive and memory CD4^+^ T cells from 8 children with vertically acquired HIV who were on ART for a median of 8 years were quantified and characterized. We consistently detected HIV in naive CD4^+^ T cell–sorted collections at frequencies that exceeded the upper bounds of possible infected memory cell contaminants with 95% statistical confidence. Frequencies of naive CD4^+^ T cell infection varied among participants and were markedly lower than those determined for memory CD4^+^ T cells, in agreement with previously reported measurements from adult samples (26, 56–58). However, naive cells comprised the majority of the CD4^+^ T cell pool and were consequently found to substantially contribute to the HIV reservoir as previously reported in pediatric nonhuman primate models of SIV/SHIV infection (58, 59). As expected, we found that age was strongly negatively correlated with the circulating levels of naive CD4^+^ T cells (frequency and absolute count) as well as with their contribution to the HIV reservoir. The frequency of infection of naive CD4^+^ T cells from participant 1001 was the highest of all participants, representing 40% of the total infected peripheral CD4^+^ T cells in this donor. It may be that variations in naive CD4^+^ T cell numbers, lineages, and fractional contribution to total peripheral T cells between the time of infection and ART administration contribute to differences in these values among participants, even after years on ART. Moreover, the mode of vertical transmission might have a pronounced effect on early infection dynamics that is perpetuated longitudinally. For instance, direct HIV transmission to the fetal circulatory system in utero might introduce the virus to a T cell population composed almost entirely of fetal naive cells having a distinctive lineage and differing capacities relative to older those of children and adults (21–24), whereas postnatal exposure through breastfeeding might instead introduce the virus to a developing gut enriched with early memory cells (60). Regardless, the relative abundance of HIV^+^ naive CD4^+^ T cells in samples from participant 1001 allowed us to deeply characterize proviruses obtained from this participant.

By screening for HIV genetic elements Psi and RRE in a modified IPDA (42), we found a mean frequency of 4.7% potentially intact proviruses in naive CD4^+^ T cells. The intactness of 1 provirus from participant 1001 was confirmed by segmental PCR amplification and sequencing. The frequencies of intact proviral DNA we report here in naive CD4^+^ T cells, ranging from less than 1% to 8.8%, are generally consistent with frequencies reported in total CD4^+^ T cells for adults initiating ART during acute or chronic infection and for children initiated on ART early in life estimated using near-full-length sequencing methods (37, 44). Little data exist regarding the frequency of intact proviruses within naive CD4^+^ T cells. One study limited to 2 adults living with HIV initiated on ART during the chronic phase of HIV infection reported 3.3% and 6.6% of intact proviruses in naive CD4^+^ T cells after 9 years of ART (41). Another report from the same group shows notably variable levels of intact proviruses, 0.9% to 17% after 5–6 years of ART, in the naive CD4^+^ T cells from 5 adult patients with chronic progression of the disease who were specifically selected for their large range of reservoir sizes (61). Generally low-to-undetectable levels of intact proviruses were also found in 2 groups of 5 adults treated early or late during chronic HIV infection (62).

Despite the finding that naive T cells typically express CCR5 at low levels (63–65), we found that all proviruses harbored in naive CD4^+^ T cells from multiple participants in this cohort were predicted to be R5 tropic, in agreement with previous coreceptor usage predictions for naive CD4^+^ T cell proviruses in adults (41). Moreover, higher infection frequencies, such as we observed in naive cell samples from participant 1001, may result from transiently elevated CCR5 expression caused by various stimuli (35, 66, 67) and perhaps coinciding with expansive virus replication early in infection. Certain conditions can enhance HIV infection of naive CD4^+^ T cells, including transmission in a tonsil lymphoid tissue microenvironment ex vivo (68, 69) or in coculture with monocyte-derived DCs (MDDCs) in vitro (70), although the latter condition was only shown to enhance infection by CXCR4-tropic (X4), but not CCR5-tropic (R5), virus. Of particular note with respect to this study, preincubation with IL-7, a cytokine critical for naive T cell homeostatic proliferation and survival (71), has been shown to sensitize umbilical cord blood naive T cells, but not adult naive T cells, to HIV infection (72–74). Although further study is required, it is tempting to speculate that elevated IL-7 levels in infancy or later childhood may have contributed to the relatively high frequencies of both infection and clonal expansion reported for naive CD4^+^ T cells of participant 1001.

Naive CD4^+^ T cells display distinct characteristics that may affect HIV reservoir dynamics. Naive cells have long half-lives and a slow turnover rate as compared with other maturational T cell subsets and, as such, exhibit the slowest HIV DNA decay over time, typically affected more by differentiation into memory cells than by cell death (75). Here, HIV ISA allowed us to assess the degree to which specific clonal populations were expanded in the naive and memory CD4^+^ T cell subsets of participant 1001. Samples from this participant proved to be exceptional in this regard as well, with large clonal populations harboring proviruses integrated into *TSEN34* and *HLA-A* projected to comprise 7.2% and 14.6% of HIV^+^ peripheral naive and memory CD4^+^ T cell subsets, respectively, at the time of sample collection. Indeed, detection of clonally infected cell populations in either subset more than once at this level of sampling is indicative of a high degree of clonal expansion, as was the case for 13.4% of naive and 25% of memory CD4^+^ T cell integration sites from this donor. Although the frequencies of clonal expansion measured here were quite high, relative levels between the 2 subsets were consistent with reports indicating that the degree of naive T cell clonal expansion, driven by homeostatic mechanisms, is typically less than that of memory cells driven by both antigen responses and homeostatic proliferation (41, 76). However, determining absolute clone sizes or sizes relative to uninfected cell populations would require a broad TCR characterization of much larger naive and memory CD4^+^ T cell samples.

We analyzed proviral integration sites and relative expression levels of genic integration targets for evidence of differentiation of infected naive T cells into memory CD4^+^ T cells between the times of infection and sample collection. The most direct evidence of this having occurred would be the identification of an integration site common to sorted naive and memory CD4^+^ T cells, but this was not observed, even for the largest expanded clones characterized by integration into *TSEN34* and *HLA-A*. Although we observed integrations into *CHD2* and *LOC339788* genes in both naive and memory CD4^+^ T cells, the integrations occurred at distinct positions in these genes, and thus neither was indicative of differentiation of clonally expanded infected naive T cell populations. A prior analysis of HIV integration sites in naive and memory CD4^+^ T cell subsets in adults found rare instances of identical integration sites only in the memory cell populations, suggestive of linear differentiation from infected central memory cells into effector memory cells (77). This result contrasts with a previous study in adults that reported common proviral sequences between naive and subsets of memory CD4^+^ T cells (41). It is important to note that not identifying clonal population of HIV^+^ CD4^+^ T cells in both naive and memory sorted collections does not mean that differentiation of infected naive cells into memory does not occur. Indeed, we should perhaps expect such observations to be rare, even exceedingly so, since identification of an HIV^+^ clone in both subsets would only be favored if it had become highly expanded prior to antigen-mediated activation, was overrepresented relative to other memory cell clones afterwards, and both subpopulations remained elevated at the time of sample collection. 

HIV preferentially integrates into transcriptionally active genes (49, 50), and ART effectively suppresses ongoing viral replication (78). One implication of these now well-established precepts is that integration sites identified in individuals on ART have the potential to serve as indicators of the transcriptional state of cells at the time of infection, as well as how drug, fitness, and immune-selective pressures shape the pool of persistent infected cells over time on ART. Considering the levels of gene expression and integration site frequency for the first time in segregated naive and memory cells obtained from a CLWH, we found that nearly 4 of 5 integration site genes identified in naive cells were more highly expressed in this subset relative to memory cells. The converse was true for the 5 most highly expressed integration site genes identified in HIV^+^ memory cells, with HLA-A being the integration site for the largest clone of memory CD4^+^ T cells as well as the most highly expressed gene in both memory and naive cells. These patterns both support our conclusion that most HIV^+^ cells in the sorted naive cell collection are not memory cell contaminants and reveal relative gene expression as a useful comparative metric of subset-selective integration site preferences.

Among the specific integration site genes identified in naive CD4^+^ T cells, *BACH2* and *IL2RB* are particularly worthy of note, as both have been reported to confer a survival advantage to infected T cells (51, 52). We identified 3 independent integration sites in *BACH2* among naive CD4^+^ T cells of participant 1001 — the only integration site gene meriting this distinction. Moreover, all integrations in *BACH2* were found within introns and in the same orientation as gene transcription. *BACH2* expression levels were more than 4-fold higher in naive CD4^+^ T cells than in memory T cells, and this gene has specifically been shown to maintain T cells in a naive state by suppressing effector memory–related genes (79). Taken together, these data support the notion that the survival advantage conferred by HIV integration into BACH2 may be naive cell selective. Unlike *BACH2*, *IL2RB* is clearly associated with T cell memory function, encoding the β subunit of the high-affinity form of the receptor (IL-2R) for IL-2, a cytokine that mediates activated CD4^+^ T cell development into memory T cells, among other functions (80, 81). Moreover, relative *IL2RB* expression in memory CD4^+^ T cells exceeded that in naive CD4^+^ T cells by almost 3-fold. Surprisingly, the *IL2RB* integration site was identified twice in naive CD4^+^ T cells, indicative of a clonally expanded T cell population, but not in memory CD4^+^ T cells. It must be noted, however, that the site in naive cells was scored as gene proximal (i.e., 3,176 base pairs outside of the gene), not in an intron, as the proposed mechanism for conferring a survival advantage would require. Though anecdotal, our results suggest that the capacity for integration into these genes to confer a survival advantage may be selective for naive or memory T cell subtypes.

It is important to note that approximately 91% of proviruses contributing to the naive T cell integration site profile of participant 1001 were found to be defective (IPDA^–^) and not part of a viral reservoir capable of kindling viremic rebound when ART is withdrawn. Furthermore, most naive cell integration sites were identified near or within expressed genes,which is consistent with this observation, as intact HIV proviruses that persist in individuals on long-term ART have been reported to be mostly integrated in nongenic, pericentromeric, or other regions of the genome considered repressive to gene expression (82–87). Whether the distinctive integration site profiles of persistent reservoir cells can be further dissected to reveal patterns specific to naive cells, or even naive cells in children who acquired HIV at birth, should be a priority subject for future studies.

In this work, we provide evidence for the persistence of HIV proviruses in naive CD4^+^ T cells in children with perinatally acquired HIV on long-term ART. Frequencies of genetically intact proviruses in this subset resemble those previously reported for children and adults, providing evidence that naive cells contribute to the HIV reservoir in children. Our findings also indicate that large HIV^+^ naive CD4^+^ T cell clones were not predisposed toward activation, proliferation, and/or differentiation into memory T cells, indicating that they may be a more challenging target for latency reversal cure strategies. Collectively, these results serve as a foundation for future studies that, with larger and longitudinal sampling, may allow us to further distinguish subtype-specific and generalized characteristics of infection in PLWH at different ages and phases of immune development.

## Methods

### Sex as a biological variable.

Our study evaluated samples from male and female children with HIV, and similar findings are reported for both sexes.

### Naive and memory CD4^+^ T cell isolation by FACS.

PBMCs were isolated by density-gradient centrifugation, and CD4^+^ T cells were negatively selected from freshly isolated PBMCs using magnetically labeled microbeads and subsequent column purification according to the manufacturer’s protocol (Miltenyi Biotec). Enriched peripheral CD4^+^ T cells were rested overnight in RPMI–20% FBS and then stained with previously determined volumes of the following fluorescently conjugated monoclonal antibodies: CD3-AF700 (clone SP34-2), CCR7-FITC (clone 150503), CD8-APC-Cy7 (clone SK1), CD45RA-PE-Cy7 (clone 5H9), CD45RO-APC (clone UCHL1), and CD27-PE (clone M-T271) from BD Biosciences; CD28-PE-Cy5.5 (clone CD28.2) from Beckman Coulter; and CD4-BV650 (clone OKT4) and CD95-BV605 (clone DX2) from BioLegend. Circulating cell populations were defined as follows: naive CD4^+^, CD45RO^–^CD45RA^+^CD28^+^CD27^+^CCR7^+^CD95^–^ and memory CD4^+^, CD45RO^+^CD95^+^. Sorting was performed on a FACSAria LSR II (BD Biosciences) equipped with FACSDiva software.

### Multiple displacement amplification of single proviruses.

MDA was performed as previously described (40) with minor modifications. Genomic DNA was extracted from collections of sorted naive and memory T cells and partitioned across 96- or 384-well plates so that less than 30% of the wells contained a single provirus. DNA in individual wells was chemically denatured and neutralized, after which the remaining reaction components were added to the final concentrations indicated: 1× Phi29 DNA pol reaction buffer (New England Biolabs [NEB]), 50 mM KCl, 1 mM dNTPs, 200 mM trehalose, 50 μM random DNA pentamer (5N) or hexamer (6N), and 0.25 U/μL NEB Phi29 DNA polymerase. Final reaction volumes were scaled to 40 μL and 10 μL for 96- and 384-well plates, respectively. Reactions were incubated at 30°C (6N primer) or 28°C (5N) for 18 hours, and then for 10 minutes at 65°C to inactivate the polymerase.

### HIV LTR, Psi, and RRE detection in MDA products.

Aliquots of 1:3 MDA product dilutions in 5 mM Tris, pH 8, were used for sequential screening, first for HIV LTR, and then for Psi and RRE, by singleplex and multiplex real-time PCR, respectively. Dedicated sets of primers and hydrolysis probes and 2× Roche LightCycler 480 Probes Master mix were utilized in these reactions in accordance with the manufacturer’s instructions. Psi and RRE primers and probes (Supplemental Table 3A) were adapted from the IPDA (42). The purpose behind the slight modification of the primer and probe sequences, together with an increase in the number of PCR cycles from 50 to 55, was to maximize assay sensitivity to and tolerance for minor variation in target sequences, including across HIV subtypes. Amplifying from MDA wells rather than single-copy proviruses in genomic DNA was also advantageous in these respects.

### Calculating the probability of contaminating memory CD4^+^ T cells in the naive CD4^+^ T cell subset.

The sizes and purities of the naive and memory T cell populations and the observed numbers of infected cells in each participant were used to determine the number of possible memory T cell contaminants within naive T cell subsets (Supplemental Schema 1). Briefly, the total number of infected cells is the sum of the fraction of naive cells that are infected (fN) times the number of naive cells, plus the fraction of memory cells that are infected (fM) times the number of memory cells. From this equation for each subset, fN and fM are determined, where fM times the number of memory cells in the naive population gives the expected number of infected cells arising from the memory cells. Poisson distribution is used to determine the 95% probability that the number of infected memory cells in the respective infected naive T cell counts is Nmax or less.

 Equation 1


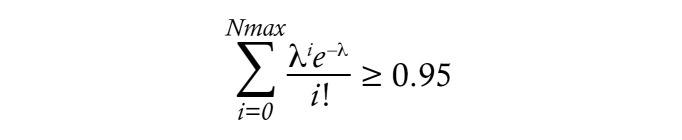


These values were in turn used to determine the minimum number infected naive cells in the infected naive T cell counts.

### Determination of intactness of HIV proviruses by segmental PCR and long-read sequencing.

In participant 1001, proviruses in naive T cell MDA reactions that screened positive for LTR, Psi, and RRE were reamplified by nested or double-nested PCR in segments using the primers listed in Supplemental Table 3B. Using the HXB2 sequence as a reference, primers were designed for nested PCR of 4 overlapping segments (Q1–Q4) with expected outer/inner amplicon sizes of 2,145/2,056, 2,524/2,399, 2,922/2,841, and 1,947/1,876 bp, respectively. Inner nested PCR products visible by agarose gel electrophoresis and of appropriate size were sequenced. To obtain Q1–Q4 segments that failed to amplify, MDA products or outer Q1–Q4 PCR amplicons were reamplified in smaller subsegments of Q1–Q4, dubbed D1–D10 (where D1 and D2 comprise Q1; D3, D4 and D5, comprise Q2; D6, D7, and D8 comprise Q3; and D9 and D10 comprise Q4). Full coverage was achieved for products of 1 naive T cell MDA reaction of participant 1001. Overlapping amplicons were individually subjected to Sanger sequencing and pooled for construction of an Oxford Nanopore Technologies (ONT) sequencing library using the LSK114 ligation sequencing kit. ONT sequencing was performed in a Flongle Flow Cell (R10.4.1) on a MinION Mk1C instrument and base-called using the “superaccuracy” model (SUP) in Guppy, version 6.5.7, software. A near-full length provirus contig was assembled from ONT sequences, and Sanger sequences were overlaid for sequence validation and to fix ambiguous base calls using Geneious Prime 2020.2.4 software. The completed assembly was submitted for analysis using the proviral sequence annotation and intactness test (ProSeq-IT) (45) and was determined to be sequence intact.

### Phylogenetic analysis of intact provirus identified in naive CD4^+^ T cells.

Neighbor-joining phylogenetic analysis was used to ensure that the intact sequence detected in participant 1001’s naive T cells was not the result of laboratory or cross-sample contamination. The HIV LANL Sequence Database was accessed to obtain pregenerated full-length genome alignments. Subtype B sequences that were dated between 2000 and 2019 were randomly sampled and globally distributed (*n* = 29) (Supplemental Table 3). Additionally, 3 sequences were selected as they were previously amplified products in our laboratory (88) (donor C02, MT744340; donor F07, MT745572; donor R09, MT745575). To rule out potential laboratory strain contamination, NL4.3 (AF324493.2), JRCSF (M38429.1), 89.6 (U39362.2), YU2 (M93258.1), LAI (MN691959.1), and HXB2/LAV (K03455.1) were included in the analysis. A near full-length alignment including subtype B reference sequences, laboratory strains, and the predicted intact proviral sequence from participant 1001 were aligned with MAFFT, version 7.450 (FFT-NS-1 200PAM/k=2 algorithm; ref. 89) in Geneious Prime 2020.2.4. Minor manual adjustments were performed as needed. Two regions were phylogenetically analyzed: U3 and *pro-pol* for their known strong phylogenetic signal. The U3 region was from the near full-length alignment following the polypurine tract through the CATATA box (HXB2 coordinates: 9,086–9,517 bp). The *pro-pol* region was from HXB2 coordinates 2,253–5,096 bp with part of the highly variable p6 *gag* region excluded. In both analyses, a p-distance neighboring tree with complete deletions (where sites containing alignment gaps are removed prior to analysis) were reconstructed using MEGA X with bootstrapping (*n* = 10,000 pseudoreplicates) (90). The trees were visualized using FigTree software and were rooted on the NL4.3 laboratory strain.

### Determination of genotypic coreceptor usage.

To determine the coreceptor fusion specificity of proviruses found in naive CD4^+^ T cells, the V3 loop of HIV-1 *env* was amplified by PCR from a subset of MDA-amplified proviruses in naive T cells using the primer pairs described in Supplemental Table 3C. PCR reactions contained the following components: 1.25X Platinum II PCR Buffer, 0.25 mM dNTPs, 0.25 μM primer F, 0.25 μM primer R, Platinum II Taq Enzyme (Invitrogen, Thermo Fisher Scientific) and molecular-grade water, and the resulting amplicons were subjected to Sanger sequencing from the same primers used for PCR. Two separate coreceptor usage prediction algorithms were used for the codon-aligned V3 sequences: PSSM (SINSI and X4R5 matrices) (47) and Geno2Pheno (48). For the Geno2Pheno algorithm, the false positivity rate (FPR) was set to a value of 10% standard cutoff, meaning there was a 10% probability of classifying an R5 virus incorrectly as an X4 virus. As standards when running PSSM (SINSI and X4R5 matrices) and Geno2Pheno, the following reference HIV-1 isolates with the V3 sequence were used: IIIB/LAV (X4, K03455.1), 89.6 (X4, U39362.2), JR-CSF (R5, M38429.1), and YU-2 (R5, M93258.1).

### ISA.

ISA was performed on individual or pooled MDA reaction products from participant 1001, who screened positive for the HIV LTR as previously described (40, 52, 91). Briefly, bead-purified MDA products were subjected to random DNA shearing, end repair, A-tailing, and adapter ligation, which adds a known sequence to fragment termini and thus enables PCR amplification of unknown host-virus junctions. Adapters also contain functional elements required for Illumina sequencing and indexes required for multiplexing, and eventual demultiplexing, of sequencing libraries. Provirus-adjacent host-virus junction sequences were selectively amplified from ligated MDA product fragments by duplex PCR using donor-specific primers complementary to the sense U3 and antisense U5 components of the HIV LTR, together with an adapter-specific primer. Amplicon libraries were purified, quantified, and then sequenced on an Illumina MiSeq. Donor-specific ISA primer sequences are listed in Supplemental Table 3D, and comprehensive library preparation protocols and analytical workflows for the determination and characterization of HIV integration sites are provided in previously published works (40, 91). All integration sites identified and characterized in this study have been submitted to the RID (53).

### Datasets used to determine subtype-specific expression of naive and memory T cell integration site genes.

Expression levels of genes found to harbor sites of HIV-1 integration in naive and memory CD4^+^ T cells in samples from participant 1001 were determined using standard RNA-Seq reference data from sorted cells from 8 adults with HIV on ART that were previously published (55) and provided by Eli Boritz (Vaccine Research Center [VRC], National Institute of Allergy and Infectious Diseases [NIAID], NIH). The gating strategies and data processing methodologies are described again here. Stained samples were sorted into CD4^+^ T cell subsets using the BD FACSAria system by first gating for single cells that were CD3^+^, Aqua^lo^, and negative for CD11c, CD14, CD20, CD56, and TCR-γδ. The remaining cells that were CD4^+^ and CD8^–^ were then collected as naive (CD27^+^CD45RO^–^), central memory (CD27^+^CCR7^+^CD45RO^+^ or CD27^+^CCR7^–^CD45RO^+^), or effector memory (CD27^–^) CD4^+^ T cell subsets. Sorted cell subsets were processed for total RNA extraction and whole-transcriptome sequencing as previously described (92). TPM values calculated for naive, central memory, and effector memory CD4^+^ T cell subsets were averaged across donors and subsets, and weighted averages were calculated for memory T cells from central and effector memory averages in accordance with PBMC CD4^+^ T cell subtype distribution estimates recently reported for PLWH on ART (75).

### Statistics.

The Wilcoxon matched-pairs, signed-rank test was used to determine *P* values in Figure 1, Figure 2A, and Supplemental Figure 3. Spearman correlations (2-tailed) were calculated for the values reported in Figure 3. More specialized methods for calculating the probability of contaminating memory CD4^+^ T cells in the naive CD4^+^ T cell subset and determining genotypic coreceptor usage are described in dedicated sections above and in Supplemental Schema 1. For all pertinent statistical analyses, a *P* value less than 0.05 was considered significant.

### Study approval.

All samples analyzed in this study were obtained with the written consent of the guardians of the participants, using a protocol approved by the Emory University Institutional Review Board (IRB00009146) as part of the Center for AIDS Research (CFAR) specimen repository study.

### Data availability.

Raw data used to produce the graphs in Figures 1–5 are provided in the Supporting Data Values file. All HIV sequences were submitted to GenBank and assigned the accession numbers PV031991–PV031999. Integration sites are available in the RID (https://rid.ncifcrf.gov) (53) under the identifiers rid44048262_1 to rid44048262_189 or via https://rid.cancer.gov/ (53) using the PMID 40048262.

## Author contributions

MGK performed experiments and wrote the manuscript. VN performed experiments and wrote the manuscript. SG performed experiments. SCP performed experiments and bioinformatics analyses. WW performed experiments. BL performed statistical analyses. AAC performed bioinformatics analyses. XW supervised experiments and the bioinformatics analyses. AMH performed experiments. JWR performed experiments, supervised experiments, performed bioinformatics analyses, acquired funding, and wrote the manuscript. AC conceived of the idea for this study, acquired samples and funding, and wrote the manuscript. MM conceived of the idea for this study, acquired funding, performed experiments and analyses, and wrote the manuscript. MFK conceived of the idea for this study, acquired funding, supervised experiments, and wrote the manuscript. All authors critically reviewed and approved the submitted version of the manuscript.

## Supplementary Material

Supplemental data

ICMJE disclosure forms

Supporting data values

## Figures and Tables

**Table 1:**
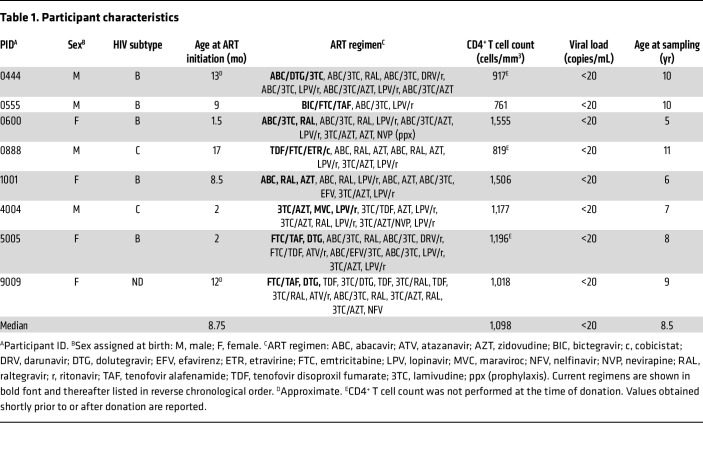
Participant characteristics

**Table 2:**
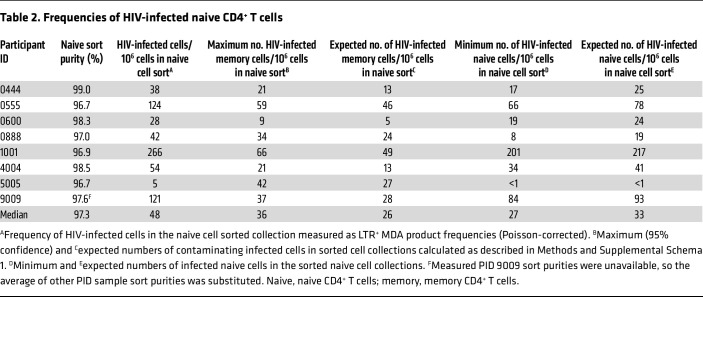
Frequencies of HIV-infected naive CD4^+^ T cells

## Version 1 03/06/2025

In-Press Preview

## Version 2 05/01/2025

Electronic publication
