# Supplementary material for: Divergent populations of HIV-infected naive and memory CD4+ T cell clones in children on antiretroviral therapy
Source: J Clin Invest. 2025 Mar 6;135(9):e188533. doi: 10.1172/JCI188533 (PMC12043081; doi:10.1172/JCI188533)
Supplement: Supplemental data [file jci-135-188533-s205.pdf]

Supplementary Tables

Table S1. Frequencies of HIV-infected memory CD4+ T cells.

Participant ID	Memory sort purity (%)	HIV-infected cells/10^6 cells in memory sort[B]	Maximum # of HIV-infected naïve cells/10^6 cells in memory sort[C]	Expected # of HIV-infected naïve cells/10^6 cells in memory sort[D]	Minimum # of HIV-infected memory cells/10^6 cells in memory sort[E]	Expected # of HIV-infected memory cells/per 10^6 cells in memory sort[F]
**0444**	100.0	1,251	0	0	1,251	1,251
**0555**	97.4	1,368	9	2	1,359	1,366
**0600**	94.6	252	7	1	245	251
**0888**	93.0	729	9	1	720	728
**1001**	96.9	1,546	15	7	1,531	1,539
**4004**	96.3	799	7	2	792	797
**5005**	97.4	810	0	0	810	810
**9009**	96.5[A]	1,143	9	3	1,134	1,140
Median	***97.3***	***977***	***8***	***2***	***972***	***975***

[A] Measured PID 9009 sort purities were unavailable, so average of other PID sample sort purities was substituted. [B]Frequency of infection of sorted populations of naïve and memory CD4+ T cells measured by HIV LTR PCR in MDA well and Poisson-corrected. [C]Maximum (95% confidence) and [D]expected numbers of contaminating infected cells in sorted cell collections calculated as described in Materials and Methods and Supplementary Data Schema S2. [E]Minimum and [F]expected numbers of infected cells in the sorted subset. Naïve: naïve CD4+ T cells; memory: memory CD4+ T cells.

Table S2. Sequences accessed from Genbank for comparative phylogenetic analysis

Isolate	Country	Year	Accession
B.AR.00.85891FL_2000.KY968403	Argentina	2000	KY968403
B.AR.03.03AR138910.DQ383749	Argentina	2003	DQ383749
B.AR.09.DEMB09AR010.MH078530	Argentina	2009	MH078530
B.AR.10.DEMB10AR006.KY658684	Argentina	2010	KY658684
B.AR.14.DEMB14AR012.MH078531	Argentina	2014	MH078531
B.AR.15.DEMB15AR013.MH078532	Argentina	2015	MH078532
B.BE.16.STAR10_TCM_TTM_A1_85.MZ041446	Belgium	2016	MZ041446
B.BE.17.02006_cen.MN449474	Belgium	2017	MN449474
B.BG.08.V_08_001.MH746261	Bulgaria	2008	MH746261
B.BR.10.DEMB10BR038.KU749390	Brazil	2010	KU749390
B.CA.19.P3_G12_CD200R1.MW881698	Canada	2019	MW881698
B.CN.07.SX070080.JF932493	China	2007	JF932493
B.ES.10.R6.KT200355	Spain	2010	KT200355
B.FR.09.DEMB09FR001.KF716494	France	2009	KF716494
B.GB.11.MM34.23.1B2.QVOA.MT794569	United Kingdom	2011	MT794569
B.GE.03.03GEMZ010.DQ207942	Georgia	2003	DQ207942
B.JP.05.DR6737.AB287364	Japan	2005	AB287364
B.KR.08.KR021.MT021909	South Korea	2008	MT021909
B.PE.07.FAM193.MN887109	Peru	2007	MN887109
B.PH.15.DEMB15PH003.KY658690	Philippines	2015	KY658690
B.PH.16.1022.MH327757	Philippines	2016	MH327757
B.RU.09.09RU4457.JX500709	Russia	2009	JX500709
B.SE.15.101SE.MF373203	Sweden	2015	MF373203
B.TH.13.2544072P000FL_Sd.MG989506	Thailand	2013	MG989506
B.US.05.CR0208W.FJ469706	United States	2005	FJ469706
B.US.15.C02.02_20181218CADHFH_MATR3.MT744340	United States	2015	MT744340
B.US.15.F07.02_20190325CADHFH_ZNF721.MT745572	United States	2015	MT745572
B.US.15.R09.01_20190805CADHFH_ABCA11P.MT745575	United States	2015	MT745575
B.US.17.UNC_367_PL1_I17.MW062703	United States	2017	MW062703
B.YE.02.02YE507.AY795904	Yemen	2002	AY795904
B.YE.02.02YE508.AY795905	Yemen	2002	AY795905
B.ZA.09.DEMB09ZA022.KP109515	South Africa	2009	KP109515

Tables S3. Oligonucleotides used in this study

S3A. Primers and probes used to detect HIV LTR, Psi, and RRE in MDA wells.

Primer Name	5' Modification	Sequence	3' Modification
LTR 517+	None	CTTAAGCCTCAATAAAGCTTGCC	None
LTR 577-	None	GGATCTCTAGTTACCAGAGTC	None
LTR probe HEX, PT	/5HEX/	AGTAGTGTG/ZEN/TGCCCGTCTG	/3IABkFQ/
Psi 695+	None	GACTCGGCTTGCTGAAG	None
Psi 775-	None	CATCTCTCTCCTTCTAGC	None
Psi probe FAM, PT	/56-FAM/	TTTTGGCGT/ZEN/ACTCACCAGT	/3IABkFQ/
RRE 7722+	None	GCAGAGAGAAAAAAGAGC	None
RRE 7812-	None	GCCTGTACCGTCAGC	None
RRE probe HEX, PT	/5HEX/	TTCCTTGGG/ZEN/TTCTTGGGAGCAG	/3IABkFQ/

S3B. Primers used for segmental amplification of PID:1001 intact provirus

Primer Name	Sequence	Outermost position (hxb2)
B cons D1F, Q1F, P1F	CTTAAGCCTCAATAAAGCTTGCC	518
LTR,F-Q1-F2	AGTAGTGTGTGCCCGTCTG	552
B cons D1R	GTCTACATAGTCTCTAAAGGGTTC	1683
B cons D2F	AGCAGGAACTACTAGTACCCTTC	1497
Pol,R-Q1-R2	TTGGGCCATCCATTCCTGGCTT	2607
B cons D2R, Q1R, P1R	CAAATTTCTACTAATGCTTTTAT	2662
B cons D3F, Q2F, P2F	GCACTTTAAATTTTCCCATTAGTCCTA	2536
Pol,F-Q2-F2	AAGCCAGGAATGGATGGCCCAA	2586
B cons D3R	CTGTTTTCTGCCAGTTCTAGCTCT	3469
B cons D4F	CTGCCAGAAAAAGACAGCT	3285
B cons D4R, P2R	GGTTAAAATCACTAGCCATTGCTCTC	4311
B cons D5F, P3F	CAGTTAATAAAAAAGGAAAAGGTCTAC	4119
IN,R-Q2-R2	TGTATTACTACTGCCCCTTCACCTTT	4984
B cons D5R, Q2R	CCTGCCATCTGTTTTCCA	5059
B cons D6F, Q3F	TTTGGAAAGGACCAGCAAA	4930
IN,F-Q3-F2	AAAGGTGAAGGGGCAGTAGTAATACA	4959
B cons D6R, P3R	TCCGCTTCTTCCTGCCATAG	5987
B cons D7F, P4F	ATTATTCGACAGAGGAGAGCAA	5806
B cons D7R	TGTACATTGTACTGTGCTGACATT	6968
B cons D8F	CCAATAGATAATAATAATACTAGCTATAGGT	6771
Env,R-Q3-R2	TGCTCCCAAGAACCCAAGGA	7799
B cons D8R, Q3R, P4R	GTCTGGCCTGTACCGTCAGC	7851
B cons D9F, Q4F, P5F	AGTGGTGCAGAGAGAAAAAAGAGC	7736
Env,F-Q4-F2	TCCTTGGGTTCTTGGGAGCA	7780
B cons D9R	CCATCTTATAGCAAAGCCCTT	8800
B cons D10F	CTGCAGTATTGGAGTCAGGAACTA	8622
LTR,R-Q4-R2	CAGACGGGCACACACTACT	9655
B cons D10R, Q4R, P5R	GGATCTCTAGTTACCAGAGTC	9682

S3C. Primers used for determining genotypic co-receptor usage.

Primer Name	Sequence	Application
E20F	GGGCCACACATGCCTGTGTACCCACAG	Outer (PCR1) Amplicon: 938bp
E115R	AGAAAAATTCCCCTCCACAATTAA	Outer (PCR1) Amplicon: 938bp
E30F	GTGTACCCACAGACCCCAGCCCACAAG	Inner (PCR2) Amplicon: 887bp
E125R	CAATTTCTGGGTCCCCTCCTGAGG	Inner (PCR2) Amplicon: 887 bp

S3D. Donor specific ISA linker and primer sequences.

Oligo name	Oligo sequence
TlinkerUMIlong	AGTTCAGACGTGTGCTCTTCCGATCTATTACTCGNNNNNNNNNNTAGTGCTCCGCTTAGAGGACT
TlinkerUMIshort	/5Phos/GTCCTCTAAGCGGAGCACTA/3AmMO/
LinkerPrimer1	AGTTCAGACGTGTGCTCTTC
LinkerPrimer2	CAAGCAGAAGACGGCATACGAGATATTACTCGGTGACTGGAGTTCAGACGTGTGCTCTTCCGATC
HIV3LTRPrimer1	CTGGTACTAGAGATCCCTCAGA
HIV3LTRPrimer2	AATGATACGGCGACCACCGAGATCTACACCCTATCCTACACTCTTTCCCTACACGACGCTCTTCCGATCTNNNNNN<br>TAGCGAGTCCTTTTAGTCAGTGTGGAAAATC
HIV5LTRPrimer1	GTGTGGTAGACCCACAGATCAAGGA
HIV5LTRPrimer2	AATGATACGGCGACCACCGAGATCTACACGGCTCTGAACACTCTTTCCCTACACGACGCTCTTCCGATCTNNNNNN<br>CTGCGTGTTGTCTTTTCTGGGACCAAACTAGCC

Color Coding & Oligo Modifications

Inline-Index
UMI
I7 Index
I5 Index
Illumina Diversity
Illumina Primer
Viral Sequence
Double-Stranded Linker Complementary Sequence

/5Phos/	5'-phosphate
/3AmMO/	3'-amino modifier

Supplementary Schema

Schema S1. Calculating the probability of contaminating CD4+ memory T cells in the CD4+ naïve T subset. To determine the number of possible memory cell contaminants within naïve subset, several factors were considered. Based on HIV LTR values in both the naïve and memory populations and the percent purity of each population, the framework can begin where the number of infected cells in both naïve and memory populations can be determined based on the observed. By determining the fraction of infected cells of both populations, the expected memory cell population can be calculated and treated as the variance given a Poisson distribution with 95% confidence of the memory population as described below:

Given the following parameters:

T_N: Total number of cells examined in the Naïve subset.
I_N: Total number of infected cells in the Naïve subset.
P_N: Purity of the naïve cells in the Naïve subset.
N_N: Number of naïve cells in the Naïve subset (= P_N*T_N)
N_M: Number of memory cells in the Naïve subset (= $T_N – N_N$)
T_M: Total number of cells examined in the Memory subset.
I_M: Total number of infected cells in the Memory subset.
P_M: Purity of the naïve cells in the Memory subset.
M_M: Number of memory cells in the Memory subset (= P_M*T_M)
M_N: Number of naïve cells in the Memory subset (= $T_M – M_M$)
F_N: Fraction of naïve cells that are infected.
F_M: Fraction of the memory cells that are infected.

The total number of infected cells in the Naïve subset is:

$$I_N = F_N * N_N + F_M * N_M$$

Similarly, the total number of infected cells in the Memory subset is:

$$I_M = F_N * M_N + F_M * M_M$$

These two equations are then used to determine F_N and F_M. The expected number of infections from memory cells in the Naïve subset is:

$$\lambda = F_M * N_M$$

This expected number is the mean that one would expect from multiple trials. To determine the upper 95% confidence limit, we use a Poisson distribution and determine the number N_{max} where:

$$\sum_{i=0}^{Nmax} \frac{\lambda^i e^{-\lambda}}{i!} \geq 0.95$$

The minimum number of infections of naïve cells in the Naïve subset is then:

$$I_N – N_{max}$$

Supplementary Figures

Figure S1.

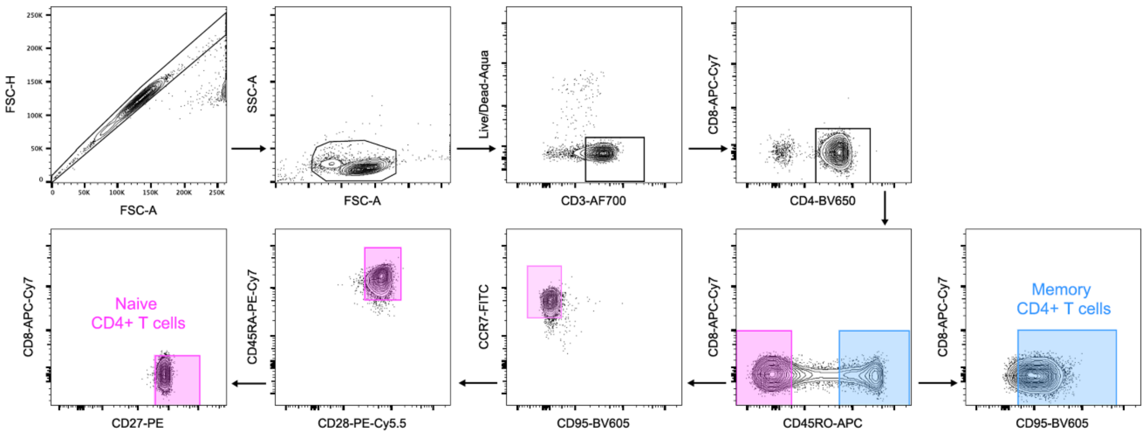

Gating strategy for the cell sorting of CD4+ T cell subsets. Representative fluorescence-activated cell sorting (FACS) dot plots showing the gating strategy used for the cell sorting of peripheral blood naïve and memory CD4+ T cells from CLWH.

Figure S2.

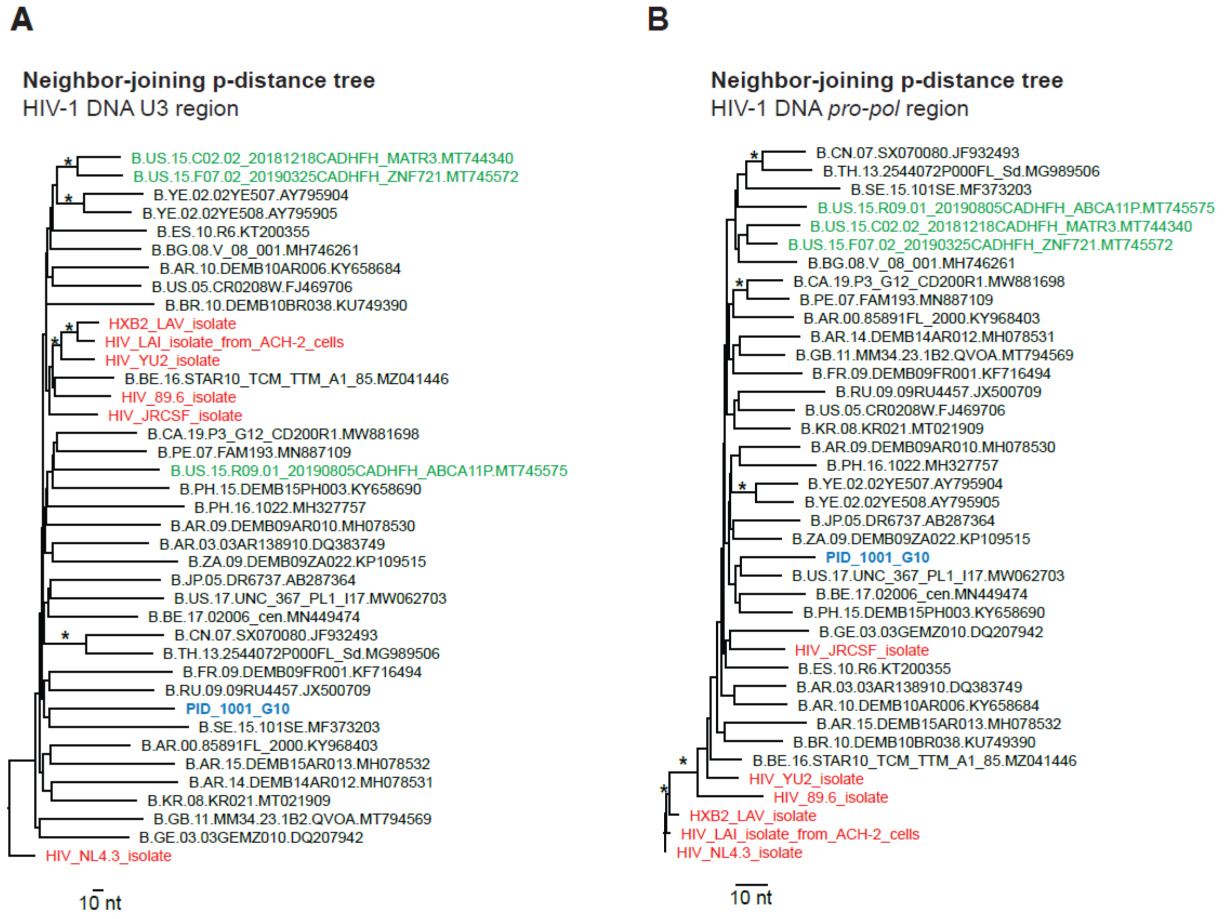

Phylogenetic analyses demonstrate that the intact HIV provirus detected in the naïve CD4+ T cell subset is not a laboratory contaminant. (A) Neighbor-joining p-distance tree of the HIV-1 DNA U3 region for reference sequences, laboratory strains, and intact provirus of PID 1001. **(B)** Neighbor-joining p-distance tree of HIV-1 DNA *pro-pol* gene for reference sequences, laboratory strains, and intact provirus of PID 1001. Sequences are colored with PID 1001 intact provirus (bold blue), laboratory strains (red), previously NFL-SGS amplicons isolated in our laboratory (green), and subtype B reference sequences (black). The scale is set to 10 nt. Bootstrap support >70% (*).

Figure S3.

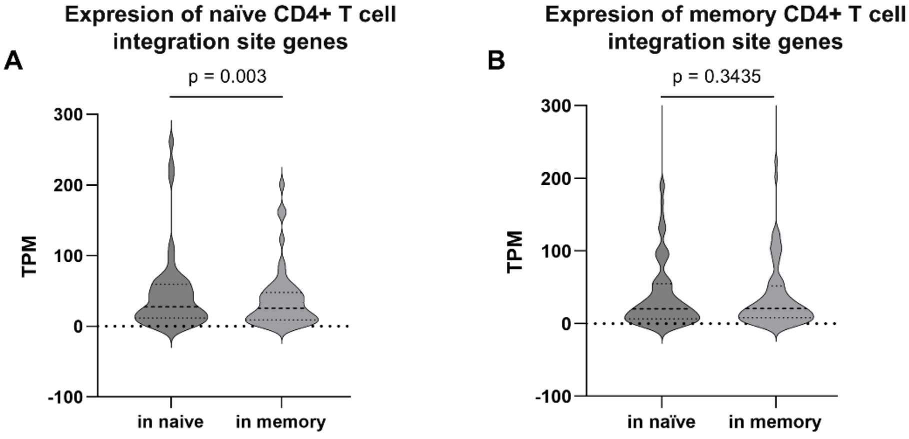

Expression of integration site genes in naïve and memory CD4+ T cell subsets. (A) Expression of naïve CD4+ T cell integration site genes in naïve and memory CD4+ T cells. Median, 1st quartile, and 3rd quartile expression levels are indicated. **(B)** Expression of memory CD4+ T cell integration site genes in naïve and memory CD4+ T cells. Median, 1st quartile, and 3rd quartile expression levels are indicated. Y-axes are capped at 300 TPM for clarity, and P values calculated from Wilcoxon matched-pairs signed rank tests for the respective comparisons are indicated.
